# Characteristics of 1738 Patients With Coronavirus Disease 2019 (COVID-19) in Wuhan, China

**DOI:** 10.1017/dmp.2021.129

**Published:** 2021-04-30

**Authors:** Xiao Ma, Yujing Gao, Lingling Di, Hanning Ma, Bin Mei, Junjian Zhang, Amei Wang, Ke Feng, Lishan Yang, Zhongwei Chen

**Affiliations:** 1 Department of Emergency, General Hospital of Ningxia Medical University, Yinchuan, China; 2 Department of Biochemistry and Molecular Biology, School of Basic Medical Sciences, Ningxia Medical University, Yinchuan, China; 3 Department of Otorhinolaryngology, General Hospital of Ningxia Medical University, Yinchuan, China; 4 Department of Social Medical Development, Zhongnan Hospital of Wuhan University, Wuhan, China; 5 Department of Neurology, Zhongnan Hospital of Wuhan University, Wuhan, China

**Keywords:** SARS-CoV-2, coronavirus disease 2019, COVID-19, characteristics, follow-up

## Abstract

**Objective:**

Since December 2019, severe acute respiratory syndrome coronavirus 2 (SARS-CoV-2) has been discovered in Wuhan and spread rapidly across China and worldwide. Characteristics of infected patients are needed to get insight into the full spectrum of the disease.

**Methods:**

Epidemiological and clinical information of 1738 diagnosed patients during February 7-26, 2020 in Wuhan Dongxihu Fangcang Hospital were analyzed. A total of 709 patients were followed up on symptom, mental health, isolation site, and medication after discharge.

**Results:**

There were 852 males and 886 females in the cohort. The average age of the patients was 48.8 y. A total of 79.98% of the patients were from Wuhan, Hubei Province. The most common initial symptoms were fever, cough, and shortness of breath. Among all the patients, 1463 had complications, with respiratory distress as the most common complication. The average duration of hospitalization was 15.95 ± 14.69 d. The most common postdischarge symptom is cough. After discharge, most patients were full of energy and chose hotel as their self-isolation site. Coronavirus disease 2019 (COVID-19) Chinese medicine No.2 prescription is the medication used most commonly by the patients after discharge.

**Conclusions:**

The population is generally susceptible to SARS-CoV-2. After receiving aggressive treatment of combined Chinese and Western medicine, most patients had a good prognosis and mental health after discharge.

Since December 2019, a novel coronavirus pneumonia (NCP) outbreak in Wuhan occurred in China, and later spread to the rest of cities in China and many countries abroad.^1^ On January 7, 2020, an institute from China identified its pathogen as a novel coronavirus.^2^ The National Health Commission of China quickly included it in Class B infectious diseases and adopted approaches for Class A infectious diseases to prevent and control it.^3^ On January 30, 2020, World Health Organization (WHO) declared the novel coronavirus outbreak as a Public Health Emergency of International Concern (PHEIC).^4^ On February 11, 2020, WHO named the disease caused by the novel coronavirus as COVID-19.^4^ The disease was highly contagious, and the population was generally susceptible to it.^3^ As the second batch of medical teams from Ningxia Hui Autonomous Region of China, the author and colleagues assisted and treated 1738 patients in the period from February 7-26, 2020 in Dongxihu Fangcang Hospital of Wuhan. Here, we analyzed the epidemiological and clinical data, as well as the follow-up information of the patients with COVID-19 to complement the epidemiology and clinical characteristics of the outbreak.

## Methods

### Patients

The epidemiological and clinical data of 1738 patients with COVID-19 admitted to the Dongxihu Fangcang Hospital of Wuhan during February 7-26, 2020 were collected. Information including administrative area, gender, age, and initial symptoms was extracted and analyzed. Of all the patients, 846 were planned to be followed up on the symptom, mental health, isolation site, and medicine used after discharge, of which, 137 cases were lost to follow-up, and 709 cases of patients completed the follow-up. Psychological status of the patients on follow-up was categorized to full of energy, full of hope, anxiety, depressed, or frustrated according to the assessment by clinicians.

The epidemiological and clinical information, treatment methods, and imaging examination of the patients included in this article have obtained the permissions of the patients and the management department of Wuhan Dongxihu Fangcang Hospital. The study was approved by Ningxia Medical University General Hospital Scientific Research Ethics Committee.

### Diagnostic Criteria

Patients with COVID-19 were diagnosed on the basis of epidemiological history, clinical manifestations, viral nucleic acid detection, laboratory detection, and imaging examination.^5^

Epidemiology history was defined as having 1 of the following within 14 d before disease onset: (1) travel history or residence history of Wuhan City and surrounding areas, or other areas with COVID-19-infected cases reported; (2) contact history with patients infected with COVID-19; (3) patients with fever or respiratory symptoms from Wuhan and surrounding areas, or from areas where COVID-19-infected cases have been reported; (4) aggregated onset. Two or more cases with fever and/or respiratory symptoms in a small area, such as home, office, or school class.

Clinical manifestations included fever and/or respiratory symptoms.

Viral nucleic acid in respiratory secretion samples standardly taken by throat swab was detected by reverse transcriptase-polymerase chain reaction (RT-PCR). All patients in this cohort had positive RT-PCR results for severe acute respiratory syndrome coronavirus 2 (SARS-CoV-2).

**Figure 1.**
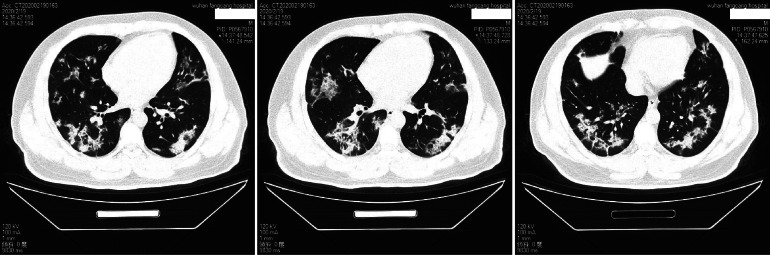
Chest CT of a patient with COVID-19. A typical CT manifestation of 40-y-old male patient, showing ground glass-like exudation in both lungs.

Imaging diagnosis is 1 of the diagnostic criteria for COVID-19, and chest computed tomography (CT) scan is 1 of the criteria. Chest CT is, therefore, a definite diagnosis and a screening program. Especially after the outbreak of the pandemic, all patients with related respiratory symptoms were asked to do chest CT examination to avoid misdiagnosis, on the premise of excluding contraindications and obtaining permissions from patients. Most patients had CT of chest before admission. CT was performed again at least 1 wk after hospitalization, and compared with the previous CT manifestations to assess the patients’ condition and the therapeutic effects. Figure 1 is a typical CT manifestation of 40-y-old male patient, showing a ground glass-like exudation.

### Treatment

General treatment included: (1) bed rest, ensuring sufficient heat, maintaining water and electrolyte acid-base balance, monitor vital signs, and blood oxygen saturation, and so on; (2) timely and effective oxygen therapy for patients, including nasal catheter, mask oxygen, and nasal high flow oxygen therapy; (3) Arbidor was used for antiviral therapy (for adult, the dose is 200 mg/time, 3 times/d). Nucleic acid detection was performed after 10-d of Arbidor treatment; if the result is positive, then continue the treatment. Traditional Chinese Medicine Lianhua Qingwen capsules were used as an adjuvant therapy (4 capsules/time, 3 times/d). Patients can take oseltamivir or ribavirin orally outside of hospital. (4) Patients with bacterial infection in the lungs were given moxifloxacin orally or intravenously for antibiotic therapy.

COVID-19 Chinese medicine No.2 prescription^6^ is the traditional Chinese medicine used for treatment. The compositions of it for basic prescription are Maxing Shigan Decoction, Tingli Dazao Xiefei Decoction, Huopu Xialing Decoction, Shenshu San, and Dayuanyin. Drug compositions of it include 6 g of Raw Ephedra, 15 g of Raw Gypsum, 9 g of Almond, 15 g of Qiang Huo, 15 g of Tinglizi, 9 g of Guanzhong, 15 g of Dilong, 15 g of Xu Changqing, 15 g of Huoxiang, 9 g of Pei Lan, 15 g of Atractylodes, 45 g of Yunling, 30 g of Raw Atractylodes, 15 g of Jiao Sanxian, 15 g of Magnolia officinalis, 9 g of Jiao Betel nut, 9 g of Simmering grass fruit, and 15 g of Ginger.

### Statistical Analysis

Descriptive statistical analysis was applied in this study. Continuous variables, such as age and duration of hospitalization, were presented as mean and standard deviation. Categorical variables were summarized as counts and percentages. Patients lost to follow-up were not included in the analysis of follow-up data.

## Results

### Epidemiological Characteristics

From February 7-26, 2020, Wuhan Dongxihu Fangcang Hospital admitted 1738 patients, all of them were common type.^7^ Among all the patients, there were 852 males and 886 females, with a ratio of 1 to 0.96. The patients range in age from 4 to 90, with an average age of 48.80 ± 12.73 y old. Of all the patients, 0.75% (13 of 1738) were younger than 18 y old, and 9.44% (164 of 1738) were older than 65 y old.

The patients were from Wuhan and other areas of Hubei Province, and other provinces throughout China (Table 1). Among all the patients, 1390 patients (79.98%) came from Wuhan City, of which 342 were from Dongxihu District, accounting for 19.68% of all the patients and 24.60% of patients from Wuhan City, making Dongxihu the district with the highest proportion of patients in the 13 administrative districts of Wuhan.

**Table 1.** Regional distribution of 1738 patients with COVID-19

Region	Cases *n*(%)	Region	Cases *n* (%)
**Wuhan, Hubei**	1390(79.98)	**Other provinces** **in China**	124(7.13)
Dongxihu District	342(19.68)	Henan	32(1.84)
Jiang’an District	271(15.59)	Guangdong	11(0.63)
Wuchang District	204(11.74)	Hunan	10(0.58)
Qiaokou District	163(9.38)	Jiangsu	10(0.58)
HongshanDistrict	114(6.56)	Sichuan	9(0.52)
Jianghan District	105(6.04)	Hebei	8(0.46)
Qingshan District	64(3.68)	Jiangxi	7(0.40)
Hanyang District	58(3.34)	Anhui	7(0.40)
Huangpi District	33(1.90)	Gansu	7(0.40)
Jiangxia District	11(0.63)	Heilongjiang	2(0.11)
Caidian District	9(0.52)	Xinjiang	6(0.35)
Hannan District	8(0.46)	Tianjin	5(0.29)
Xinzhou District	8(0.46)	Guizhou	4(0.23)
**Other cities in Hubei**	224(12.89)	Shandong	4(0.23)
Xiaogan	80(4.60)	Chongqing	2(0.11)
Jingzhou	33(1.90)		
Huanggang	32(1.84)		
Xiangyang	31(1.78)		
Shiyan	18(1.04)		
Huangshi	17(0.98)		
Ezhou	13(0.75)		

A total of 224 patients were from other areas of Hubei, accounting for 12.89% of total patients. Among them, 80 patients were from Xiaogan City, Hubei Province, accounting for 4.60% of the total patients, making Xiaogan the city with the highest proportion of patients except Wuhan in Hubei. Ezhou had the lowest proportion of patients in Hubei Province, with 13 cases (0.75%) in total.

A total of 124 patients were from other 15 provinces in China, accounting for 7.13% of all the patients. Most of these patients (32 cases) were from Henan Province, accounting for 1.84% of the total number of patients, and 25.81% of the patients from provinces other than Hubei; the fewest number of patients were from Chongqing and Heilongjiang, only 2 cases in each province, accounting for 1.61% of the patients from provinces other than Hubei.

### Clinical Characteristics

The initial symptoms included fever, cough, chest tightness, shortness of breath, muscle aches, fatigue, diarrhea, sore throat, dizziness, and so on (Table 2). The most 3 common initial symptoms were fever, cough, and shortness of breath, with 712 (40.97%), 503 (28.94%), and 347(19.97%) patients, respectively. The duration of fever is 1 to 30 d, with an average of 11.04 d. The number of patients with dizziness as initial symptom was the fewest (12 cases, accounting for 0.69% of all the patients). Of note, 4 patients were asymptomatic and hospitalized due to positive nucleic acid test during disease screening.

**Table 2.** Initial symptoms and complications of the patients with COVID-19

Initial symptom	Cases (*N* = 1738)	Complications	Cases (*N* = 1463)
Fever, *n* (%)	712(40.97)	Respiratory distress, *n*(%)	813(55.57)
Cough, *n* (%)	503(28.94)	Headache, *n*(%)	197(13.47)
Shortness of breath, *n*(%)	347(19.97)	Vomiting, *n*(%)	186(12.71)
Muscle ache, *n*(%)	67(3.86)	Chill, *n*(%)	81(5.54)
Fatigue, *n*(%)	47(2.70)	Stomach ache, *n*(%)	70(4.79)
Diarrhea, *n*(%)	26(1.49)	Stuffy nose, *n*(%)	58(3.96)
Sore throat, *n*(%)	24(1.38)	Palpitations, *n*(%)	35(2.39)
Dizziness, *n*(%)	12(0.69)	Hoarse voice, *n*(%)	23(1.57)

Among all the patients, there were 1463 cases with coexisting symptoms on admission. Complications included nasal congestion, chills, respiratory distress, nausea and vomiting, palpitations, hoarseness, abdominal pain, and headache. The most common complications were respiratory distress, headache, nausea, and vomiting, which in 813 patients (55.57%), 197 patients (13.47%), and 186 patients (12.71%), respectively. The number of patients with concurrent hoarseness was the fewest (23 cases, accounting for 1.57% of the patients).

### Patient Follow-up

All patients were cured and discharged or transferred to designated hospitals after effective treatment, and no deaths. The average duration of hospitalization was 15.95 ± 14.69 d. The patients took medications orally after discharge according to the doctors’ instructions and could contact the doctor at any time. Within 2 wk after discharge from the hospital, patients were followed up by telephone for 3 times on 7, 10, and 14 d after discharge. The follow-up information included clinical symptoms, mental health, isolation site, and medications (Table 3).

**Table 3.** Follow-up information of 709 patients with COVID-19

Follow-up contents	Time after discharge		
	7 days	10 days	14 days
**Symptom**			
Fever, *n*(%)	1(0.14)	1(0.14)	10(0.00)
Cough, *n*(%)	46(6.49)	46(6.49)	46(6.49)
Shortness of breath, *n*(%)	19(2.68)	20(2.82)	20(2.82)
Muscle ache, *n*(%)	2(0.28)	3(0.42)	2(0.28)
Fatigue, *n*(%)	4(0.56)	3(0.42)	3(0.42)
Diarrhea, *n*(%)	6(0.85)	6(0.85)	6(0.85)
Sore throat, *n*(%)	6(0.85)	5(0.71)	5(0.71)
Nausea, *n*(%)	2(0.28)	2(0.28)	0(0.00)
**Mental state**			
Full of hope, *n*(%)	36(5.08)	36(5.08)	35(4.94)
Full of energy, *n*(%)	659(92.95)	658(92.81)	659(92.95)
Anxiety, *n*(%)	10(1.41)	10(1.41)	11(1.55)
Frustrated and depressed, *n*(%)	4(0.56)	5(0.71)	4(0.56)
**Isolation site**			
Home, *n*(%)	137(19.32)	139(19.60)	139(19.60)
Hotel, *n*(%)	362(51.06)	360(50.78)	360(50.78)
Community, *n*(%)	23(3.24)	23(3.24)	23(3.24)
School, *n*(%)	187(26.38)	187(26.38)	187(26.38)
**Medication**			
Lianhua Qingwen capsule, *n*(%)	7(0.99)	7(0.99)	7(0.99)
Jin Lianhua capsule, *n*(%)	1(0.14)	1(0.14)	0(0.00)
COVID-19 Chinese medicine no.2 prescription, *n*(%)	125(17.63)	125(17.63)	125(17.63)
Antibiotic, *n*(%)	3(0.42)	3(0.42)	2(0.28)
Antiviral, *n*(%)	2(0.28)	1(0.14)	1(0.14)

A total of 846 cases of patients were planned to be followed up, of which 137 patients were lost to follow-up, and the final number of patients followed up is 709, including 348 males and 361 females, with an average age of 45.15 ± 12.64 y old. The reasons for being lost to follow-up were refusing to answer the call, error information, and null phone number.

A small number of patients still had intermittent symptoms after discharge from the hospital. The main symptoms after discharge were the initial symptoms. The most common postdischarge symptom was cough, with 46 cases in each time of the 3 follow-ups, accounting for 6.49% of the total follow-up patients; and the least common postdischarge symptom was fever (1, 1, and 0 cases in the 3 follow-ups, respectively).

Mental health was divided into 4 types: full of energy, full of hope, anxiety, and depressed and frustrated. The majority of patients were full of energy after discharge (659, 658, and 659 cases in the 3 follow-ups, respectively). The number of patients feeling depressed and frustrated was the fewest (4, 5, and 4 cases, respectively).

Home, hotel, community, and school are the main places chosen by patients for isolation after discharge. Among them, most patients chose hotel for self-isolation (362, 360, and 360 cases in the 3 follow-ups, respectively); and the place with the least chosen is community (23 cases in each time of the 3 follow-ups).

All patients in our study were treated with Western medicines and traditional Chinese medicines, especially COVID-19 Chinese medicine No.2 prescription, during hospitalization. The main function of this prescription was to improve respiratory symptoms. Other oral drugs, such as Lianhua Qingwen capsule, were used as adjuvant drugs. Patients with intermittent mild respiratory symptoms on discharge were advised to continue taking oral medications after discharge until the symptoms disappeared, such as Lianhua Qingwen capsule, Jin lianhuan capsule, COVID-19 Chinese medicine No.2 prescription, antibiotics, and antiviral drugs. Among them, most of the patients chose COVID-19 Chinese medicine No.2 prescription for further treatment at home (125 cases in each of the 3 follow-ups, accounting for 17.63% of the actual follow-up patients), and the Jin lianhuan capsules was the least chosen by the patients (1, 1, and 0 case in the 3 follow-ups, respectively).

## Discussion

Coronavirus is a type of single stranded positive strand RNA virus with an envelope. Epidemiological studies have shown that the main transmission route of SARS-CoV-2 is close contact, which occurs from person to person through droplets, aerosols, and contact.^1,7^ Quite a few patients have symptoms that are atypical or even are asymptomatic during the incubation period of infection, becoming a potential source of infection for SARS-CoV-2,^8^ which is also in line with the characteristic of extremely rapid spread in the initial stage of the outbreak.

One of the characteristics of COVID-19 is that it is highly infectious and transmitted among people. Hence, the hospitalization of patients can reduce the mass transmission and control the number of patients from the source to avoid causing a panic. In addition, because the disease has a latent period, centralized hospitalization is an effective approach to isolate and observe the patients, and administer timely and effective treatment once being diagnosed. From February 7 to 26, 2020, a total of 1738 patients with COVID-19 were admitted to the Dongxihu Fangcang Hospital of Wuhan. Among them, 852 cases were male and 886 cases were female, with a ratio of 1:0.96. As for population distribution, the youngest patient was 4 y old and the oldest patient was 90 y old; patients of all ages were susceptible to the virus, indicating age is not a factor determines whether individuals can be infected by SARS-CoV-2 or not, which is consistent with the recent reports.^9–11^

The Dongxihu Fangcang Hospital is located in the Dongxihu District of Wuhan City. It was temporarily transformed from a cultural center to that of a hospital for the admission of COVID-19 patients, with 6416 beds in 3 halls. The patients admitted were from 13 of administrative districts in Wuhan (1390 patients, accounting for 79.98% of the total patients), 7 of other cities in Hubei (224 patients, accounting for 12.89%), and 15 of other provinces in China (124 patients, accounting for 7.13%). The wide distribution of the patients may be due to the increased number of people traveling between cities, owing to the traditional Chinese Spring Festival. When the outbreak occurred, considering the long incubation period and rapid transmission of COVID-19, the principle of nearby isolation and quarantine, nearest diagnosis and treatment were adopted to achieve the purpose of early detection, early isolation and quarantine, and early treatment, thereby reducing the clustering occurrence of the disease to some extent, and saving time for the treatment of severe patients as well.

The most common initial symptoms in our cohort were fever, cough, chest tightness, and shortness of breath. The most common complication is respiratory distress. This result is similar to the characteristics of patients hospitalized in the epidemic areas.^12,13^ Clinically, we found that the virus causes lung lesions first, resulting in obvious symptoms in the respiratory system, which is consistent with Wang and colleges’ report that the chest CT findings in 138 patients diagnosed with COVID-19 exhibit bilateral patchy shadows or ground-glass opacity.^12^

The follow-up information of 709 patients indicates that the most common postdischarge symptom is cough; fever is extremely rare. Patient with intermittent symptoms on discharge took oral medication, and kept in contact with the doctors in charge. A total of 92.95% of all the follow-up patients were full of energy, and few of them suffered from anxiety or frustration. Considering that they were isolated and away from family and relatives, and experienced the entire process of the illness, it is inevitable of the occurrence of mood swings and other discomforts; and our doctors also did humane care and psychological counseling for them. For the patients who were lost to follow-up, we assigned special personnel responsible for the isolated sites, provided regular on-site services, encouraged family and friends to communicate with them through various ways to strengthen psychological counseling for them, to safely get them through the time of post-charge isolation.

All patients in our cohort were treated with traditional Chinese medicines and Western medicines as well; and Chinese medicines played a crucial role in the treatment, especially COVID-19 Chinese medicine No.2 prescription. Over the years, traditional Chinese medicines have shown potent preventive effects against viral diseases, and attracted worldwide attention and affirmation for its unique efficacy, especially for the treatment of SARS in 2003.^14^ Experts generally believe that traditional Chinese medicine functions through the following mechanisms^15,16^: (1) early use for mild patients can quickly reduce fever and relieve symptoms; (2) preventing the deterioration of the disease condition; (3) reducing the mortality of severe patients; (4) adjusting the patient’s immune system, promoting antiviral immune recovery in the body, and favoring recovery from the disease.

The epidemic occurred in Wuhan, which belongs to the Yangtze River Basin, a region with several rivers and lakes. At that time, it was an abnormal warm winter and often rainy, providing a suitable environment for long-term surviving in the air of the virus, and increasing the probability of viral transmission among people.^17^

There are some limitations and deficiencies in our study. First, a large number of patients were admitted in various medical centers distributed in Wuhan after the outbreak. Our sample size is not large enough; hence, the interpretation of the findings may be limited by it. Second, because the hospital was rebuilt temporarily, the electronic medical record system was incomplete, and the patients’ laboratory examinations were in paper form and were not included in this study. Third, because this is a retrospective study, some data are missing due to the heavy labor intensity of medical staff during the special period of outbreak, which may affect the quality of this article.

## Conclusions

From this study, we concluded that, in the early diagnosis of patients, it is necessary to inquire about the epidemiological history in detail and strictly follow the national diagnosis and treatment scheme, and confirm the diagnosis as soon as possible by combining clinical symptoms, laboratory indicators, and imaging examinations. Combination of traditional Chinese medicine with Western medicine is a more effective treatment choice for COVID-19; and psychological counseling and humanistic care are also very necessary. The 4-early principle, including early detection, early reporting, early isolation and quarantine, and early treatment, is the effective approach to control the outbreak.

## Data Availability

The datasets used and/or analyzed during the current study are available from the corresponding author on reasonable request.
